# Concentration of gambling spending by product type: analysis of gambling accounts records in Norway

**DOI:** 10.1080/16066359.2024.2340456

**Published:** 2024-04-19

**Authors:** Ingeborg Rossow, Viktorija Kesaite, Ståle Pallesen, Heather Wardle

**Affiliations:** a Norwegian Institute of Public Health, Oslo, Norway; b School of Social & Political Sciences, University of Glasgow, Glasgow, Scotland; c Department of Psychosocial Science, University of Bergen, Bergen, Norway

**Keywords:** Gambling, losses, distribution, concentration, tracking data, Norway

## Abstract

**Background:**

Most previous studies on the distribution of gambling losses were based on self-reported data. In this study, we employed tracking data (i.e. electronic betting records) to examine the concentration of gambling losses and whether concentration varies by product type.

**Method:**

Tracking data were provided by the Norwegian gambling monopolist, Norsk Tipping (NT). Data comprised of 14 different games for a random draw of 2% (*N* = 39 995) of all NT’s customers in 2019. We applied three measures of concentration of gambling losses: the mean to median ratio, the Gini coefficient, and the proportion of total losses accounted for by the upper 1%, 5% or 10% of those who gamble.

**Results:**

Across the 14 games, the mean/median ratio was 2.22, ranging from 1.37 to 17.48 for the different games, whereas the overall Gini coefficient was 0.65, ranging from 0.55 to 0.90. The upper 1%, 5% and 10% of those who gamble accounted for 17.9% (range = 5.6 − 3 8.3%), 39.5% (range = 23.6 − 74.3%), and 52.2% (range = 37.9 − 86.9%) of the losses, respectively. High concentration of losses was especially pronounced for one type of lottery (Keno), two online casino games (KongKasino and Bingoria), and for two sports betting games (Oddsen and Tipping). These findings were consistent across measures.

**Conclusion:**

Overall, the results lend strong support to the notion that a disproportionately large fraction of gambling losses are accounted for by a relatively small minority of people and that concentration of losses varies by product type.

## Introduction

There is some evidence suggesting that a substantial fraction of the gambling industry’s revenues stems from a small proportion of people who gamble, often those experiencing greater gambling harms (Fiedler et al. 2019). This evidence suggests that the distribution of gambling losses is skewed to the right and hence a few people who lose the most account for a disproportionately large fraction of total gambling losses (Tom et al. 2014). While a skewed distribution of consumption can be found for various consumer goods (e.g. nonalcoholic beverages; (Zhong et al. 2018)), it seems that ‘addictiveness’ may add to the skewness of distribution and concentration of consumption. Thus, some evidence suggests that the more addictive potential of the product, the greater the concentration of consumption (Rossow and Bramness 2015). In the present study, we examine the extent to which concentration of gambling losses varies across gambling activities that probably differ in addictive potential.

Understanding the extent to which gambling revenues are concentrated among consumers is pertinent as it provides insight into how commercial gambling entities generate their profits. Understanding whether gambling industry revenue (i.e. consumer losses) are highly concentrated among a few individuals or more equitably distributed across the fuller population of consumers provides potential indications of the relative risk and potential harm that may arise when engaging in different gambling formats. In addition, researchers have noted that those experiencing harms contribute disproportionately to industry revenues because of: a) their propensity to spend more money, more often than other consumers and, b) because for some activities (e.g. Electronic Gaming Machines, (EGMs)) those experiencing harms represent a substantial proportion of the population (Tom et al. 2014; Fiedler et al. 2019; Forrest and McHale 2022). Fiedler et al. (2019) found high levels of gambling concentration among people who display moderate risk/problem gambling behavior, suggesting that concentration of demand could be considered an additional indicator to measure the social risk of gambling markets.

There is a small, but emerging, body of evidence which has examined both the concentration of gambling revenues overall and by specific product formats. This evidence indicates a consistent pattern by which gambling expenditure is highly concentrated among a minority of individuals (see Fiedler et al. 2019 and Kesaite et al. 2023 for reviews). The few studies which have examined concentration by format have found patterns by which gambling activities such as lotteries are the least concentrated, and those with more continuous and rapid play cycles (like EGMs, online casino games/slots) are the most concentrated (Kesaite et al. 2023). With some notable exceptions, several of these studies have relied on self-reported gambling expenditure survey data, with attendant issues of measurement error and accuracy of the findings obtained. In this study, we add to this small literature by employing accurate gambling records data from Norway. Moreover, considering the strict regulations of gambling in Norway (see Table 1), it is of interest to compare gambling concentration to that in other countries with less restrictive regulations.

**Table 1.** Description of gambling products from norsk tipping by ways of delivery, content type and provider’s restrictions on gambling.

Gambling products	Delivered where	Type of content	Restrictions
**Lotteries**			
Lotto, VikingLotto, Joker, Extra, EuroJackpot	Physically (in grocery stores etc) and online	Typically numbers- based lotteries, 1-2 events per week	Overall monthly loss limit of 20 000 NOK
Keno	Physically (in grocery stores etc) and online	Number-based lottery. Draw every day	Overall monthly loss limit of 20 000 NOK
Nabolaget	Physically (in grocery stores etc) and online	Address-based lottery. Draw every day	Overall monthly loss limit of 20 000 NOK
**Sports games**			
Oddsen	Physically (in grocery stores etc) and online	Betting on outcomes of sport events and other competitions. Draw every day	Overall monthly loss limit of 20 000 NOK
Tipping	Physically (in grocery stores etc) and online	Betting on outcomes of football matches. Draw 3 times per week	Overall monthly loss limit of 20 000 NOK
**Video gaming terminal games**			
Multix	Only at physical terminal (in kiosks, gas stations, pubs, etc.)	Various casino type games, card games, games of chance. Duration between each stake is at least 3 s	Overall monthly loss limit of 20 000 NOK. Specific loss limits of 650 NOK/day and 2 700 NOK/month Mandatory break: 10 min/hour
Belago	Only at physical terminals (in bingo halls)	Various interactive games, mostly reel games. Duration between each stake is at least 3 s	Overall monthly loss limit of 20 000 NOK. Specific loss limits of 900 NOK/day, 4 400 NOK/month Mandatory break: 5 min/hour
**Online instant games**			
KongCasino	Exclusively online	Various interactive online games (e.g. reel/slot games and table games like roulette). Duration between each stake is at least 3 s	Overall monthly loss limit of 20 000 NOK/month^1^ Mandatory break: 90 sec/hour
Bingoria	Exclusively online	Various interactive online bingo related games. Duration between each stake is at least 3 s	
e-Flax	Exclusively online	Online scratch card game. Duration between each stake is at least 3 s	

^1^ As of 2019, later the limit has been reduced.

## Literature review

To set the scene for the data analyses, we review in more detail the limited existing literature on the concentration of gambling activity by format and discuss some of the common patterns that emerge, and some of the methodological issues that arise when assessing the concentration of gambling activity.

To our knowledge, only eight studies in the past decade assessed the concentration of gambling activity; four of which were based on survey methodology and self-reported gambling behavior (Orford et al. 2013; Fiedler et al. 2019; Grönroos et al. 2021; Wardle et al. 2023). There are several methodological issues that arise when using population surveys to assess high gambling spending. From the alcohol epidemiology literature, it is well established that individuals with heavy chronic drinking and others who drink excessively are typically under-represented and under-reporting occurs due to recall errors or unwillingness to report excessive alcohol consumption (Rehm et al. 2021). Similar limitations likely apply also to population surveys assessing excessive gambling behavior (Wardle et al. 2011). This might be especially problematic when trying to assess high gambling spenders, as they typically underestimate their real losses and levels of harm caused (Auer and Griffiths 2017). Furthermore, attempts to capture gambling expenditure within surveys accurately are difficult for several reasons. Consumers often do not track their expenditure fully and they may interpret the term ‘spend’ in different ways, meaning it is not always clear what data metrics are being captured and measured (Volberg et al. 2001; Wardle et al. 2011).

Notable exceptions to the aforementioned studies based on self-report data are the works of Tom et al. (2023, 2014), Deng et al. (2021) and Forrest and McHale (2022). These studies assessed online gambling expenditures using recorded data from Internet gambling service providers. A key finding from these papers were, *inter alia*, that gambling expenditure is highly concentrated i.e. a large proportion of gambling losses were attributable to a small proportion of gamblers. Specifically, in one study the top 20% of online gaming customers accounted for almost 90% of revenues (Forrest and McHale 2022). Similarly, another study found that the top 5% − 7% accounted for 80% of gambling losses (Tom et al. 2014). A study by Deng et al. (2021) observed that the top 1% of online gambling customers accounted for 30% of net losses, while a recent publication by Tom et al. (2023) found that the top 1% of subscribers to a global internet gambling operator accounted for 62% of total spend.

Across these studies, various methodologies have been employed to assess gambling concentration. Some have used standard methods for reporting dispersion in consumption while others used summary measures of inequality such as Gini coefficients (Fiedler et al. 2019; Wardle et al. 2023). One of the major limitations of using the Gini coefficient is that it assigns greater weight to differences in the middle of the consumption distribution than at the tails (Sitthiyot and Holasut 2020). In the present context, this limitation may pose difficulties since the highest spenders are concentrated at the tail of the distribution, thus we might be underestimating the level of skewness. Others, such as Tom et al. (2023), Forrest and McHale (2022), Deng et al. (2021) and Grönroos et al. (2021) have taken a different approach, estimating the proportion of total spending attributable to a small group of gamblers, for instance the top 1%, 10%, or 20% of the gamblers, respectively. A third and very simple approach, not much employed in gambling studies, is to use the mean/median ratio. With distributions skewed to the right, the mean exceeds the median, and the more skewed the distribution, the higher is the ratio (Tabor 2010).

The results from these prior studies suggest that gambling concentration varies by product type and that fast, continuous, games such as EGMs and casino games are associated with higher levels of harm than other types of activities such as lottery (Wardle et al. 2011; Kesaite et al. 2023). This exploratory study aims to contribute to this emerging evidence base by using real-time/tracking data (i.e. electronic betting records) on gambling losses to examine the extent of concentration of gambling losses in Norway and whether this concentration varies by product type.

## Data and methods

We obtained data from Norsk Tipping AS (NT), a government owned monopolist. Data were provided in 2022 upon request from the study PI (first author) at the Norwegian Institute of Public Health (NIPH). Data were provided without any restrictions or obligations to NIPH. In Norway, NT is the largest of the two gambling monopolists offering most of the legal gambling activities^1^. NT offers a variety of land-based and online gambling products (14 different types in total), including lotteries, sports games, casino games, and instant games (Table 1). It should be noted that the term ‘casino games’ often comprises a wide array of games. Regarding the online casino games (KongKasino) of Norsk Tipping, these mainly consist of slot games, but also some table games like Roulette, Blackjack and video poker.

Several restrictions pertain to gambling at NT and for specific games. Overall, there is a maximum limit for net losses across all games, which amounts to 20 000 NOK (approx. 1700 Euros) per month (customers aged 18 – 20 years have a max loss per month of 2 000 NOK or approx. 169 euros). Moreover, for some games there are lower maximum loss limits and mandatory breaks during gaming sessions (see Table 1). Registered play is mandatory for all games offered by NT, except for paper scratch cards.

### Sample

For the present study, we asked NT for individual data from a random sample of all registered customers aged 18 and over who had gambled at least once on one of NT products in 2019. The minimum legal age for gambling on NT’s products is 18 years, and all customers are required to register and provide an ID check online *via* bank ID authorization^2^. Each gambler’s activities are electronically recorded by product and date and data are stored for up to five years (Norsk Tipping AS 2023).

We chose this calendar year to avoid any impact on gambling activities due to COVID-19 restrictions (Auer et al. 2023). An analyst at NT conducted a random sample draw of 2% of all NT customers in 2019 (*N* = 2 040 000), resulting in a sample of 39 995 persons. Our sample resembled all NT customers in 2019 regarding total gambling losses; the arithmetic mean was less than 1% higher in our sample compared to all NT customers (NT analyst, personal communication).

We asked NT to provide data on gambling losses accumulated during the calendar year 2019 for each of the 14 products offered by NT (See Table 1). Gambling losses were net expenditures on gambling when winnings were subtracted. Data on gambling losses were obtained for each of the 14 games and these were summarized across all games. The latter is termed total gambling losses. For the main analysis, we employed data only for those with net losses (in Norwegian currency; NOK) across all products (*n* = 39 475) and for each product (n’s are presented in Table 2).

**Table 2.** Descriptive statistics for distributions of gambling spending by product. Net losses in NOK.

Gambling products (number of people who gamble / number of people who gamble with net losses)	% of all gamblers with net losses	% of total losses	Maximum losses	Mean losses	Median losses	Stand dev	Ratio Mean /median
**Lotteries**							
Lotto (35 498/35 498)	89.9%	26.7%	66 860	1 173	856	1 770	1.37
VikingLotto (27 283/27 283)	69.1%	16.4%	59 896	939	507	1 430	1.85
Joker (25 346/25 346)	64.2%	5.6%	12 598	344	163	466	2.11
Extra (11 615/11 615)	29.4%	4.5%	10 100	601	202	843	2.98
EuroJackpot (21 282/21 282)	53.9%	10.7%	81 569	778	314	843	2.48
Keno (3 311/3 168)	8.0%	1.7%	129 345	866	80	4 336	10.83
Nabolaget (7 296/7 152)	18.1%	2.2%	18 700	482	200	687	2.41
**Sports games**							
Oddsen (6 391/5 357)	13.6%	10.0%	240 612	2 914	320	11 025	9.11
Tipping (4 225/4 225)	10.7%	2.8%	68 840	1 042	132	3 294	7.89
**Video gaming terminal games**							
Multix (1 314/1 109)	2.8%	3.9%	32 400	5 441	1 640	7 648	3.32
Belago (619/546)	1.4%	2.9%	53 690	8 153	3 077	10 675	2.65
**Online instant games**							
KongCasino (3 267/2 827)	7.2%	9.6%	115 928	5 274	413	13 186	12.77
Bingoria (1 292/1 165)	3.0%	0.8%	94 995	1 154	66	5 279	17.48
e-Flax (7 760/7 514)	19.0%	3.6%	55 470	740	215	1 954	3.44
Total (39 995/39 475)	100 %	100 %	240 612	3 944	1 778	38 717	2.22

In a sensitivity analysis, we also included those who had net wins during the year. For these customers, we set the value to zero on net gambling losses (Supplementary Material A).

### Measures of gambling concentration

Concentration is typically seen when the distribution of consumption (or activities) is skewed to the right, and the greater the skewness to the right, the larger is the concentration of consumption among excessive consumers. Several different measures of concentration were calculated. This included estimating the ratio of mean losses to median losses as one measure of skewness (Tabor 2010) (the larger the ratio, the more skewed to the right is the distribution). Another measure of skewness calculated was the Gini coefficient. It varies between 0 (complete equality) and 1 (complete inequality), and has been used in some studies to assess the distribution of gambling consumption (e.g. (Fiedler et al. 2019; Wardle et al. 2023)). We estimated Gini coefficients using STATA Version 17. Confidence intervals were calculated using bootstrapping with resampling of *k* = 250. A final approach comprises versions of the Pareto principle; previously used by Tom et al. (2014) and Forrest and McHale (2022). In line with this approach, we estimated the proportion of net gambling losses accounted for by the most excessive gamblers; that is the upper 1%, 5% or 10% of the gamblers. All measures of concentration were calculated for each of the 14 gambling products and for total gambling across all products. In addition, we calculated the Pearson product-moment correlation coefficient between the different measures of gambling concentration.

## Results

The sample comprised more men (54.6%) than women (45.4%). Customers were evenly distributed across 10-year age groups from 26 to 75 years, whereas proportions were smaller in the youngest (18 – 25 years) and oldest (75 years or older) age groups. Most people within the sample had spent money on at least one type of lottery (Table 2). A smaller proportion had spent money on video gaming terminal games (1.4% and 2.8%), whereas sports games (10.7% and 13.6%) and instant online games (ranging from 3.0% to 19.0%) accounted for larger proportions of the sample.

The average losses per person by game format varied substantially, the casino games Belago, Multix and KongCasino stood out with higher mean losses than other games (Table 2). For all games, the mean exceeded the median. However, the ratio of mean to median varied substantially across games; from a factor less than 2 to over 15 (Table 2). This ratio was particularly high for Bingoria, KongCasino, Keno and Oddsen (Table 2), suggesting that these games have the most highly skewed distribution. These findings are in line with the results obtained using Gini coefficients (Table 3), which suggested that Bingoria, Keno, Oddsen and KongKasino were the games with the most skewed distribution of losses. To further illustrate the concentration of gambling losses, we have included a figure displaying the Lorenz curve for total net losses (Figure 1B in Supplementary Material B).

**Table 3.** Gini coefficients of gambling losses with 95% CI, by gambling product and overall.

Gambling products	Gini coefficient with 95 % CI
**Lotteries**	
Lotto (89.9%)	0.55 [0.54 to 0.55]
VikingLotto (69.1%)	0.59 [0.58 to 0.59]
Joker (64.2%)	0.60 [0.59 to 0.60]
Extra (29.4%)	0.63 [0.63 to 0.64]
EuroJackpot (53.9%)	0.65 [0.64 to 0.66]
Keno (8.0%)	0.88 [0.86 to 0.89]
Nabolaget (18.1%)	0.62 [0.62 to 0.63]
**Sports games**	
Oddsen (13.6%)	0.85 [0.84 to 0.86]
Tipping (10.7%)	0.85 [0.83 to 0.87]
**Video gaming terminal games**	
Multix (2.8%)	0.67 [0.65 to 0.69]
Belago (1.4%)	0.64 [0.62 to 0.66]
**Online instant games**	
KongCasino (7.2%)	0.83 [0.82 to 0.84]
Bingoria (2.9%)	0.90 [0.88 to 0.92]
e-Flax (19.0%)	0.72 [0.71 to 0.73]
Total	0.65 [0.65 to 0.66]

Note: Bootstrapped SE for Gini coefficient.

Finally, we calculated the proportion of gambling losses accounted for by people who lost the most; the upper 10%, 5% or 1% (i.e. those exceeding the 90^th^, 95^th^ or 99^th^ percentile of losses, respectively) for each of the gambling products (Table 4). Across all products, the upper 10% of people accounted for half of the total losses on gambling (52.2%). This proportion varied by product and was highest for Bingoria and Keno; for these games the upper 10% of people accounted for about four fifths of the net losses. Correspondingly, for Bingoria and Keno the upper 5% of people accounted for almost three quarters of the losses. The upper 1% of people accounted for a substantial proportion of losses, and particularly so for Keno, Bingoria and Oddsen.

**Table 4.** Percentile values and proportion of net losses in NOK accounted for by the upper 10% or the upper 5% or the upper 1% of people who gamble by product.

Gambling products	90 per-centile	95 per-centile	99 per-centile	Proportion by upper 10% of people	Proportion by upper 5 % of people	Proportion by upper 1 % of people
**Lotteries**						
Lotto	2 489	3 683	7 737	40.7	28.3	11.1
VikingLotto	1 875	2 982	6 088	40.2	28.4	10.5
Joker	975	1 067	1 727	37.9	23.6	8.7
Extra	1 389	2 222	3 939	44.0	27.2	8.9
EuroJackpot	1 935	2 915	5 934	47.5	32.2	12.0
Keno	1 101	3 125	17 668	84.8	73.8	38.3
Nabolaget	1 550	2 050	2 500	45.5	28.1	8.1
**Sports games**						
Oddsen	4 859	12 283	55 462	78.9	66.1	30.8
Tipping	2 406	4 957	14 512	72.2	55.9	24.3
**Video gaming terminal games**						
Multix	17 215	24 213	31 917	44.4	25.9	5.8
Belago	24 225	32 864	45 053	41.3	24.0	5.6
**Online instant games**						
KongCasino	14 537	30 949	68 767	71.8	51.8	16.3
Bingoria	1 571	5 309	24 434	86.9	74.3	35.8
e-Flax	1 777	2 998	7 651	58.9	43.6	18.9
Total	7 946	13 025	41 732	52.2	39.5	17.9

In a sensitivity analysis, we included customers with net win during the year and re-ran the analyses presented in Table 4. The findings did not alter the overall pattern of concentration across gambling products (Supplementary Material A).

Across all three indicators of concentration, we found that five games were in the top rank: Bingoria, Keno, Oddsen, KongCasino and Tipping. The indicators were strongly and positively correlated. Pearson r for correlations between the indicators are presented in Table 5.

**Table 5.** Pearson’s *r* for bivariate correlations between indicators of concentration of gambling net losses by product.

	Mean/median ratio	Proportion by upper 10%	Proportion by upper 5 %	Proportion by upper 1 %
Gini coefficient	0.91	0.98	0.94	0.87
Mean/Median ratio		0.91	0.88	0.80
Proportion by upper 10%			0.99	0.94
Proportion by upper 5 %				0.98

Note: All correlation coefficients were statistically significant (*p* < 0.001).

## Discussion

Employing accurate gambling records of all gambling activities offered by the Norwegian gambling monopolist Norsk Tipping AS, we found, in a large randomly drawn sample (*n* = 40 000) that the distribution of gambling losses is concentrated among a relatively small group of people. For several products, more than half of total net losses were attributable to the upper 5% of people gambling on these products. The extent to which this concentration occurred, varied by type of product and this variation was consistent across different measures of concentration.

Our finding of an overall concentration of gambling losses among a small group of people is in line with findings from several previous studies from other countries (Fiedler et al. 2019; Deng et al. 2021; Tom et al. 2023; Wardle et al. 2023). For instance, Grönroos et al. (2021) found that the 4.2% of people with the highest expenditures accounted for half of all gambling expenditures. A majority of these upper 4% were people at-risk or experiencing problem gambling. Our finding is also in line with a study from Norway in 2019 which showed that the 2.1% who experienced problem gambling accounted for 17% of the total turnover on legal gambling in Norway and accounted for 46% of the turnover on gambling provided by offshore/foreign gambling companies (Kristensen et al. 2022). Another illustration of the concentration of gambling expenditures is provided by Muggleton et al. (2021) who found that the upper 1% of people who had gambled spent 58% of their income on gambling.

Employing three different measures of concentration, we observed a consistent pattern across products; concentration was most prominent for two types of online instant casino and bingo games, one type of lottery and one type of sports betting. Previous studies have reported differences in gambling concentration by product, mainly showing greater concentration among continuous forms of gambling like EGMs or online slots, and less concentration among lotteries (Williams and Wood 2007; Fiedler et al. 2019). Our results are both commensurate with and different from these patterns, confirming high concentration among online casino and bingo products, but also confirming high concentration among sports betting and a lottery product.

Direct comparisons of our results with those in the current literature can be impeded for several reasons. For one, the specific gambling products that are available to customers vary across jurisdictions and across time. For instance, content of the specific products that count as lotteries may differ across studies. In this study, Keno represents a type of lottery which has a daily draw. This is a higher event frequency than the National Lottery Draw, in the UK, for example – so although both are classed as lotteries, the characteristics of these games are different. Another reason pertains to the need for comparable time frame for snapshot of the distribution, as noted by Deng et al. (2021). Some previous studies had longer (Tom et al. 2023) or shorter (Wardle et al. 2023) time frame than one year, as in the present study. Moreover, restrictions on gambling may also differ substantially across jurisdictions and hence impact direct comparability across studies.

Nevertheless, it is still of interest to look for commonalities in patterns of concentration by product characteristics across studies. In the current study, for two instant online games (Bingoria and KongCasino) we found that the upper 5% of people gambling on these products accounted for over half of gambling losses. Data from the Norwegian Gaming Authority on assessed gambling risk (Gamgard) (Cousins 2018) for each of the products offered by NT, is in line with these findings, suggesting that instant online casino games are riskier – and probably more addictive – than most lottery type games. In addition, Delfabbro et al. (2023) found that casino type online gambling products were more strongly correlated with markers of harm. However, we found a similar concentration also for two sports games (Oddsen and Tipping) and one lottery game (Keno); the upper five per cent of people accounted for over half of the losses. Moreover, the products resembling land-based EGMs (Multix and Belago) were not among the products with the highest concentration. These findings stand – at first glance – in contrast to the general notion that EGMs and other fast continuous gambling products pose the most harm risk (presumably also with regard to severe losses), whereas lotteries, produce low levels of harm (Harris and Griffiths 2018; Delfabbro and Parke 2021).

It is, however, in this context important to consider NT’s restrictions on various gambling products. In particular, the typical fast and continuous games delivered by NT, including the online instant games and the land-based EGM type products, are restricted in several important ways, both in terms of maximum losses and mandatory breaks. Restrictions on gambling losses resembles a type of individual rationing and implies that the right tail of the distribution curve is curbed. In other words, in settings where there are no such restrictions on gambling losses, it seems likely that the distribution of spending is even more skewed to the right and that the concentration of spending is even more prominent, and particularly so for games with the most addictive potential. Empirical support for this assumption is found in the study by Kristensen et al. (2022), which showed that concentration of turnover was much higher for games provided by foreign companies compared with legal games in Norway. Moreover, Tom et al. (2023) found that the upper 1% of subscribers to a global internet gambling operator accounted for 63% of total overall spend, which is clearly higher than the estimates reported in the present study, even for the most highly concentrated games.

It is therefore likely, that the pattern of concentration across gambling products in our study would have been quite different and more strongly in line with previous study findings (Kesaite et al. 2023) and general assessment of how harm risk varies by product (Harris and Griffiths 2018; Delfabbro and Parke 2021), in a scenario where these restrictions were absent. This may suggest that restrictive gambling regulations, such as those for legal games in Norway, are effective to some extent in curbing the concentration of gambling losses and thereby preventing financial and other harms. Further research on the concentration of gambling losses across various games is warranted, and particularly from jurisdictions with various regulations of gambling.

Finally, universal player-tracking systems, such as the one we have obtained data from, are valuable in several regards; for various research and monitoring purposes, for implementation of various harm reduction measures, and for evaluating harm measures *via* field trials (Newall and Swanton 2024). Currently, such systems are used by the Finnish and the Norwegian state monopolists and it is currently proposed in other jurisdictions, though the implementation of these system are uncertain (Newall and Swanton 2024). Further development and adoption of a universal player-tracking system by other jurisdictions, when implemented by an industry independent body, should be encouraged.

### Study limitations

One limitation pertains to sample representativeness. Although our sample is representative of NT’s customers and NT’s gambling activities, the sample is not necessarily representative of all people who gamble in Norway. Those who only or mainly gamble on illegal games and/or horse bets, are under-represented in our sample, although these groups are likely small, considering the wide popularity of NT’s lotteries (Pallesen et al. 2020). More important is that our study did not cover gambling losses on illegal games and horse bets, particularly as concentration of gambling losses is likely more extreme for unrestricted illegal on-line games.

Another limitation is that we did not include survey or other data on problem gambling or other harms from gambling, unlike the study by Tom et al. (2014). Thus, we could not determine the extent to which concentration of gambling is linked to problem gambling.

## Conclusion

Even in a highly regulated gambling market with registered gambling, and regulations such as upper loss limits and other costumer protection tools, we found using tracking data strong evidence that the distribution of gambling losses is skewed to the right. This implies that a minority of people who gamble account for a disproportionately large fraction of total gambling losses. It is also likely that a large proportion of this minority are also experiencing harms. Moreover, concentration of gambling losses varied across products and was higher for some games that are typically fast and continuous, despite strong regulations. Stronger consumer protection tools may benefit people across the whole distribution of losses and particularly those at most risk of harm.

## Supplementary Material

Supplemental Material

## Footnotes

1 The other monopolist (Norsk Rikstoto) only offers horse betting, while a handful of other small gambling operators offer bingos and small lotteries.

2 Registered gambling has been enforced since 2010, mainly for responsible gambling purposes.
