# Supplementary material for: Concentration of gambling spending by product type: analysis of gambling accounts records in Norway
Source: Addict Res Theory. 2024 Apr 19;33(2):114–21. doi: 10.1080/16066359.2024.2340456 (PMC11934949; doi:10.1080/16066359.2024.2340456)
Supplement: Supplemental Material [file IART_A_2340456_SM1938.pdf]

Supplemental material A

Table 4b Percentile values and proportion of net losses in NOK accounted for by the upper 10 % or the upper 5 % or the upper 1 % of people who gamble by product. Data include customers with net wins.

Gambling products	90 per-centile	95 per-centile	99 per-centile	Proportion by upper 10%	Proportion by upper 5 %	Proportion by upper 1 %
**Lotteries**						
Lotto	2 489	3 683	7 737	40.7	28.3	11.1
VikingLotto	1 875	2 982	6 088	40.2	28.4	10.5
Joker	975	1 067	1 727	37.9	23.6	8.7
Extra	1 389	2 222	3 939	44.0	27.2	8.9
EuroJackpot	1 935	2 915	5 934	47.5	32.2	12.0
Keno	1 018	2 923	17 224	85.3 (84.8)	74.6 (73.8)	39.7 (38.3)
Nabolaget	1 550	2 050	2 500	44.2 (45.5)	25.3 (28.1)	6.6 (8.1)
**Sports games**						
Oddsen	3 816	9 441	47 753	81.8 (78.9)	69.7 (66.1)	34.0 (30.8)
Tipping	2 406	4 957	14 512	72.2	55.9	24.3
**Video gaming terminal games**						
Multix	16 010	22 114	31 644	49.9 (44.4)	29.7 (25.9)	6.9 (5.8)
Belago	24 225	32 864	45 053	44.9 (41.3)	26.1 (24.0)	6.5 (5.6)
**Online instant games**						
KongCasino	14 537	30 949	68 767	75.7 (71.8)	56.1 (51.8)	18.1 (16.3)
Bingoria	1 571	5 309	24 434	88.3 (86.9)	76.0 (74.3)	37.2 (35.8)
e-Flax	1 777	2 998	7 651	59.7 (58.9)	44.3 (43.6)	19.2 (18.9)
**Total**	7 880	12 917	41 520	52.4 (52.2)	39.8 (39.5)	18.0 (17.9)

Note: Proportions in parentheses are calculated from the sub-sample of customers with net losses only, per game or overall (as presented in Table 4). Thus, for products with no customers with net win, the proportions are identical to those presented in Table 4.

For Peer Review Only

Supplemental material B

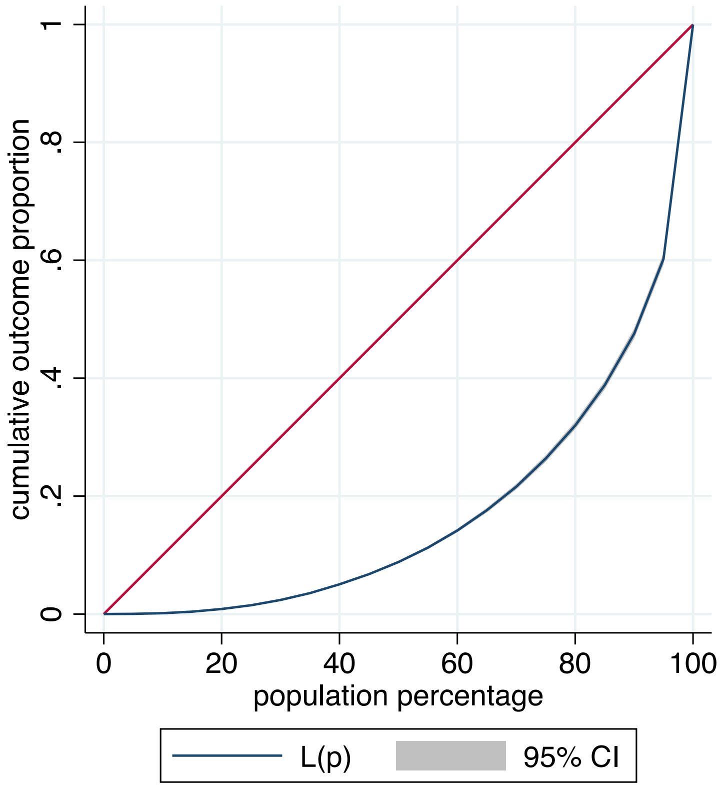

Figure 1 B. Lorenz curve, total net losses

view Only
